# The effect of cool water pack preparation on vaccine vial temperatures in refrigerators

**DOI:** 10.1016/j.vaccine.2017.11.024

**Published:** 2018-01-02

**Authors:** Geneva Goldwood, Steven Diesburg

**Affiliations:** PATH, 2201 Westlake Avenue, Suite 200, Seattle, WA 98121, USA

**Keywords:** DTP, diphtheria, tetanus, pertussis, hep B, hepatitis B, HPV, human papillomavirus, IPV, inactivated polio vaccine, MMR, measles, mumps, and rubella, OD, optical density, OPV, oral polio vaccine, PQS, Performance, Quality and Safety, WHO, World Health Organization, Cold chain, Cool water pack, Freeze-sensitive vaccine, Vaccine, Supply chain, Vaccine vial monitor

## Abstract

Cool water packs are a useful alternative to ice packs for preventing unintentional freezing of vaccines during outreach in some situations. Current guidelines recommend the use of a separate refrigerator for cooling water packs from ambient temperatures to prevent possible heat degradation of adjacent vaccine vials. To investigate whether this additional equipment is necessary, we measured the temperatures that vaccine vials were exposed to when warm water packs were placed next to vials in a refrigerator. We then calculated the effect of repeated vial exposure to those temperatures on vaccine vial monitor status to estimate the impact to the vaccine. Vials were tested in a variety of configurations, varying the number and locations of vials and water packs in the refrigerator. The calculated average percentage life lost during a month of repeated warming ranged from 20.0% to 30.3% for a category 2 (least stable) vaccine vial monitor and from 3.8% to 6.0% for a category 7 (moderate stability) vaccine vial monitor, compared to 17.0% for category 2 vaccine vial monitors and 3.1% for category 7 vaccine vial monitors at a constant 5 °C. The number of vials, number of water packs, and locations of each impacted vial warming and therefore percentage life lost, but the vaccine vial monitor category had a higher impact on the average percentage life lost than any of the other parameters. The results suggest that damage to vaccines from repeated warming over the course of a month is not certain and that cooling water packs in a refrigerator where vaccines are being stored may be a useful practice if safe procedures are established.

## 1 Introduction

Loss of vaccine due to damage from freezing is an ongoing problem. Studies from 2006 to 2015 reported that 19% of vaccine shipments in lower-income countries and 38% in higher-income countries were exposed to temperatures below recommended values [1]. Freeze damage carries two risks: that a freeze-damaged vial will be detected and therefore must be discarded, and that a freeze-damaged vial will not be detected and therefore might be administered, offering lower protection than expected. Freeze damage to a single vial can be detected using a simple shake test for aluminum-adjuvanted vaccines [2]. Wastage from freeze damage can be expensive; in 2015, freeze-sensitive vaccines worth US$1.2 billion were procured through UNICEF [1]. With the increasing use of freeze-sensitive vaccines that do not contain adjuvants and do not respond to the shake test, such as inactivated polio vaccine [3], there is increasing risk of freeze damage going unnoticed. Studies have associated vaccine exposure to freezing temperatures during transport with lower immune response [4], [5], [6]. Inadvertent freezing can occur when vaccine vials are transported in insulated carriers with ice packs to protect them from high ambient temperatures; unless the packs are partially melted first (conditioned), they can freeze adjacent vials. To combat this problem, should the commensurate reduction in cool life be acceptable, WHO recommends using water packs cooled to 2–8 °C as an alternative to conditioned ice packs for transporting freeze-sensitive vaccines. However, WHO also states that these water packs should never be cooled in a refrigerator that contains vaccines to avoid raising temperatures and compromising vaccine potency [7]. Complying with this requires a second refrigerator in clinics and health posts—an additional cost not easily absorbed in low-resource settings. While this recommendation assumes that placing warm water packs into a refrigerator is likely to damage vaccines, the thermal impact of such a practice has not been investigated. Evidence about the effect of recooling water packs alongside vaccines could enable better decisions about the need for using a separate refrigerator.

The impact of heat exposure on potency is unique to each vaccine and manufacturer. A tool that can be used to generalize this impact is the vaccine vial monitor (VVM), a heat-sensitive label required on WHO prequalified vaccines. Similar methods have been used in previous studies [8], [9] but have not been documented in detail. Each prequalified vaccine is assigned to one of four categories of VVM based on its heat stability; these are designated VVM2, VVM7, VVM14, and VVM30 (Table 1), with VVM2 vaccines being the least heat stable [10].

**Table 1:** Vaccine vial monitor (VVM) lifetime/temperature points taken from the World Health Organization (WHO) Performance, Quality and Safety (PQS) specification for VVMs. The specification requires that 90% of VVMs reach endpoint in the specified time within each specified temperature range. Examples of vaccines in each category from the WHO prequalified vaccines database and Arrhenius equations with constants calculated from the midpoint of the 90% tolerance range of WHO-supplied lifetime/temperature points are listed [10].

VVM category (vaccine examples*)	No. days to endpoint at +25 °C to +37 °C	No. days to endpoint at +22 °C to +25 °C	Time to endpoint at +2 °C to +5 °C	Arrhenius equation at midpoint of 90% tolerance range of temperatures
VVM2: least stable (OPV; some influenza)	2	NA†	225 days	$\frac{100\%}{L}=1.4422*10^{17}e^{\frac{-12\text{,}429}{T}}$
VVM7: moderate stability (IPV; MMR)	7	45	>2 years	$\frac{100\%}{L}=2.1532*10^{18}e^{\frac{-13\text{,}652}{T}}$
VVM14: medium stability (DTP; pentavalent)	14	90	>3 years	$\frac{100\%}{L}=1.0766*10^{18}e^{\frac{-13\text{,}652}{T}}$
VVM30: high stability (Hep B; HPV)	30	193	>4 years	$\frac{100\%}{L}=5.1131*10^{17}e^{\frac{-13\text{,}657}{T}}$

* Oral polio vaccine (OPV); inactivated polio vaccine (IPV); measles, mumps, and rubella (MMR); diphtheria, tetanus, pertussis (DTP); hepatitis B (hep B); human papillomavirus (HPV).

† VVM (Arrhenius) reaction rates are determined at two temperature points. WHO supplies a general range at a third point for VVM7, VVM14, and VVM30.

VVMs consist of a reference circle with a color-changing indicator dot inside. The lifetime can be defined as the time it takes for the optical density (OD) of the indicator dot to match the OD of the reference circle (referred to as the endpoint by WHO). This lifetime is temperature dependent, and there is a known relationship between temperature and lifetime for each VVM category based on the Arrhenius equation, $k=A*\exp(-E_{a}/\mathrm{RT})$. In this equation, k is the rate constant, E_a_ is the activation energy, R is the gas constant, A is a constant, and T is the temperature in kelvin [11]. The rate constant k is the bridge from lifetime versus temperature to lifetime remaining versus time: for a linear reaction, the equation for lifetime remaining versus time is y=-kt+b, where k is the rate constant from the Arrhenius equation, b is the starting lifetime, and y is the remaining lifetime. WHO specifies the lifetime at two constant temperatures for each category of VVM (Table 1). Each WHO PQS-specified lifetime-temperature point includes an implied reaction rate: 100% of the lifetime remains at 0 days; 0% of the lifetime remains at the endpoint. Because only two points are given to define the reaction rate, the reaction is assumed to be linear for currently available VVMs [12].

The goal of our study was to understand the impact on vaccine life of cooling warm water packs in a refrigerator used to store vaccines by using VVM life as a proxy for vaccine life and the Arrhenius equation for calculation of VVM life. This would give us information on the value of having a dedicated refrigerator for cooling water packs.

## 2 Materials and methods

The overall design of testing was to place water packs at 43 °C into a refrigerator with vaccine vials at 2–8 °C and measure the temperatures of the vials over time to generate warming curves. These curves were subsequently analyzed using calculated time-temperature curves for each category of VVM to estimate the impact of cyclic warming on vaccine life.

**Fig. 1:**
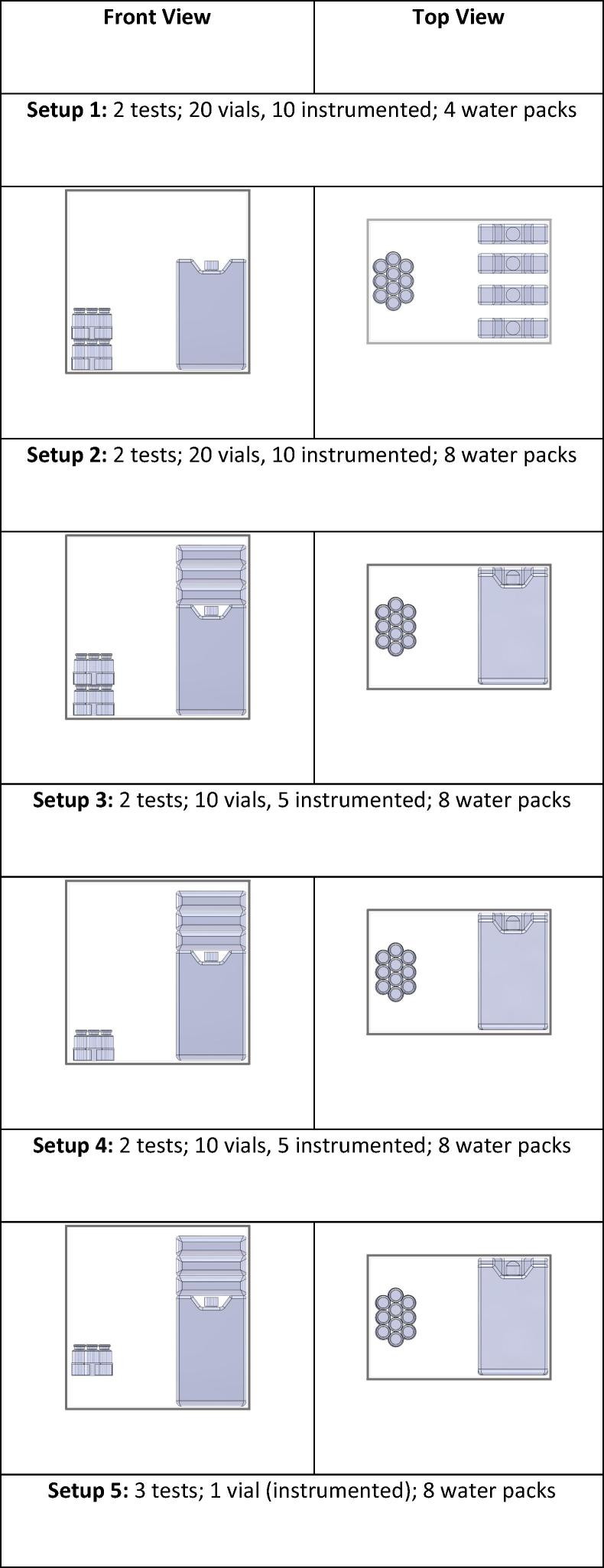

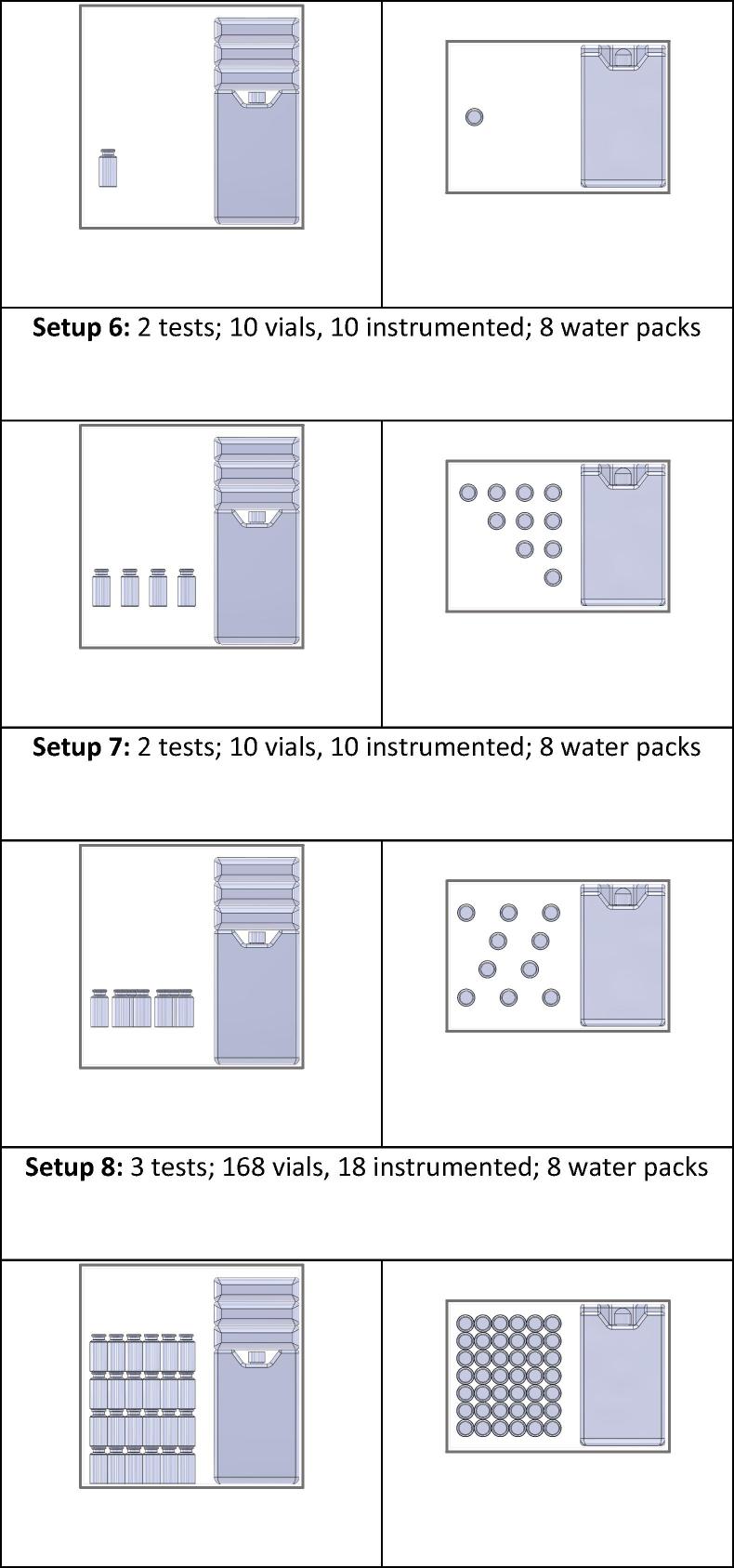
Front and top views of each test setup.

To prepare for testing, 10-mL vials were filled with water and some were instrumented with thermocouples (OMEGA Engineering, Inc., 5SRTC-TT-T-36, Stamford, CT, USA). Before the start of each test, the selected number of vials was arranged in a PQS-prequalified refrigerator (SunDanzer, model BFRV15, Tucson, AZ, USA) to cool. PQS-prequalified water packs (Blowkings, model BK 6, Mumbai, India) instrumented with thermocouples were conditioned to 43 °C, the “hot zone” temperature for prequalifying cold chain equipment [13], and placed inside the refrigerator. Several arrangements (Fig. 1) of varying numbers of vials and water packs were tested in duplicate or triplicate. Each test contained up to 18 instrumented vials; locations of these vials in each arrangement are available in Appendix 1.

Temperature collection at a rate of two samples/minute (NI cDAQ-9172 chassis, 9211 thermocouple input module, NI SignalExpress software, National Instruments Corporation, Austin, TX, USA) began immediately following placement of the water packs in the refrigerator and continued until the vials and water packs reached at least 5 °C. Warming curves were plotted and then trimmed to include only the values from the first measurement to the last one where the vial temperature exceeded 5 °C. In this way, excursion curves were generated so that a low refrigerator set point or a long test would not artificially deflate the impact of warming. A threshold of 5 °C was chosen as it is the middle of the cold chain temperature range and is a standard testing point for VVMs [14].

**Fig. 2:**
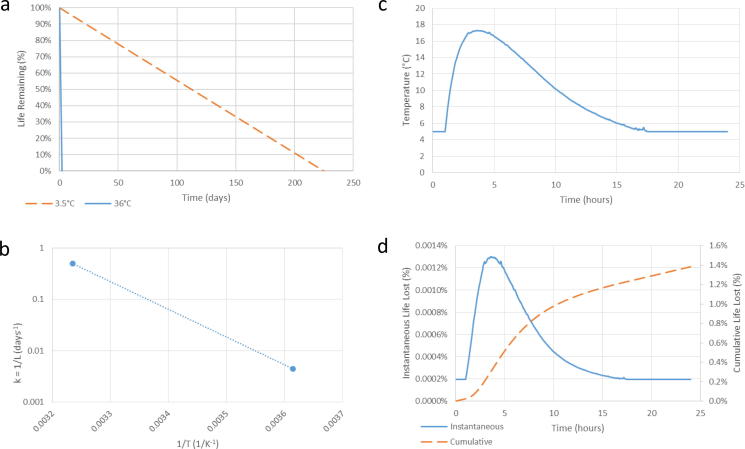
Process for calculating loss of vaccine vial monitor (VVM) life. Slopes of reaction rates at different temperatures (a), calculated from the lifetime-temperature points established by the World Health Organization, are plotted to calculate the Arrhenius curve for that VVM (b). Values shown here are for VVM2 at the center of the 90% tolerance range for reaching VVM endpoint. The calculated Arrhenius equation is applied to a warming curve from a tested vial (c) to create the instantaneous life-lost curve (d). The instantaneous values can be summed to find the cumulative life lost over 24 h (d).

Once warming curves were trimmed, an intermediate step was necessary to use VVMs to generalize the thermal impact of warming to vaccines in each category of VVM. To apply the time-temperature curves for VVMs to the measured data, it was necessary to calculate those curves from the published lifetime-temperature points. In the Arrhenius equation, $k=A*\exp(E_{a}/\mathrm{RT})$, k is the slope of the linear reaction at any given temperature (Fig. 2). Since the reaction is linear, k is equal to 100%/lifetime in days and the equation becomes $1/L=A*\exp(E_{a}/\mathrm{RT})$, where L is the lifetime. Two lifetime-temperature points are specified, leaving only two unknowns in the Arrhenius equation. Using the Arrhenius equation for each category of VVM, the reaction rate and percentage life lost during a time period at any temperature can be calculated. The Arrhenius equation for each category of VVM is shown in Table 1.

The known Arrhenius equation for each VVM must then be applied to the warming curves to estimate the impact of warming. To do this, the percentage life lost for each data point is calculated by finding the daily life lost at that temperature from the Arrhenius equation and then calculating the life lost in 30 s at that temperature. The total life lost due to warming is the sum of the values for life lost at each 30-s data point. This process is demonstrated in Fig. 2.

It is likely that vaccines would be exposed to excursions from 5 °C more than once from placement of warm water packs, but only at the last stage before use. This would be at the final health post or clinic, where cool water packs would be recooled in the vaccine refrigerator. Thus, our aim was to understand the impact of one month of repeated, daily cycling of water packs on vaccine lifetime. Because VVMs (and vaccines) continue to decay even within the cold chain, this would not be only the life lost during one excursion multiplied by 30, but rather would be life lost over 30 daily cycles, each consisting of one excursion from 5 °C plus the remainder of the day at 5 °C. This could be reported as the percentage life lost during this daily cycling, or as the amount of time the vaccines would have to be kept at a constant 5 °C for the repeated excursions to cause the VVM on the vial to reach endpoint before the end of the month. A month has been simplified here to 30 days.

## 3 Results and discussion

Average and maximum values for life lost during 30 days of cyclic warming in eight different test cases are shown in Table 2.

**Table 2:** Vaccine vial monitor life lost during 30 days of cyclic warming. Standard deviations on averages are from the pooled variance, incorporating the variation between tests and across vials. Standard deviations on maximums are for a single vial and incorporate only variation between tests. The theoretical control is the calculated life that would be lost over the applicable time period if the vaccines were kept at a constant 5 °C. See Fig. 1 for vial arrangements and Appendix 1 for instrumented vial locations.

Test case	Setup	Max vial temp	Life lost				
				VVM2	VVM7	VVM14	VVM30
Control	5 °C constant	NA	NA	16.98%	3.12%	1.56%	0.73%

1	20 vials/4 water packs	15.92 °C	Average	19.97% ± 2.50%	3.76% ± 0.55%	1.88% ± 0.27%	0.88% ± 0.13%
			Max	24.44 ± 0.34%	4.75% ± 0.08%	2.38% ± 0.04%	1.11% ± 0.02%

2	20 vials/8 water packs	17.71 °C	Average	22.90% ± 5.24%	4.64% ± 1.14%	2.32% ± 0.57%	1.08% ± 0.27%
			Max	31.77% ± 0.28%	6.61% ± 0.06%	3.30% ± 0.03%	1.54% ± 0.01%

3	10 vials on floor/8 water packs	11.48 °C	Average	19.74% ± 0.82%	3.70% ± 0.18%	1.85% ± 0.09%	0.86% ± 0.04%
			Max	21.02% ± 0.12%	3.98% ± 0.03%	1.99% ± 0.01%	0.93% ± 0.01%

4	10 vials on shelf/8 water packs	16.06 °C	Average	30.33% ± 1.43%	6.05% ± 0.33%	3.02% ± 0.16%	1.41% ± 0.08%
			Max	30.68% ± 2.42%	6.13% ± 0.56%	3.06% ± 0.28%	1.43% ± 0.13%

5	1 vial on shelf/8 water packs	17.86 °C	NA (single vial)	34.49% ± 0.28%	7.24% ± 0.06%	3.62% ± 0.03%	1.69% ± 0.01%

6	10 vials in Z-shape/8 water packs	18.02 °C	Average	28.50% ± 2.70%	5.65% ± 0.62%	2.83% ± 0.31%	1.32% ± 0.15%
			Max	32.15% ± 1.36%	6.50% ± 0.33%	3.25% ± 0.16%	1.52% ± 0.08%

7	10 vials in triangle/8 water packs	17.72 °C	Average	26.71% ± 3.35%	5.24% ± 0.76%	2.62% ± 0.38%	1.22% ± 0.18%
			Max	31.29% ± 2.52%	6.30% ± 0.59%	3.15% ± 0.29%	1.47% ± 0.14%

8	168 vials/8 water packs	17.30 °C	Average	26.06% ± 8.11%	5.10% ± 1.80%	2.55% ± 0.90%	1.19% ± 0.42%
			Max	41.49% ± 0.44%	8.56% ± 0.10%	4.28% ± 0.05%	2.00% ± 0.02%

**Fig. 3:**
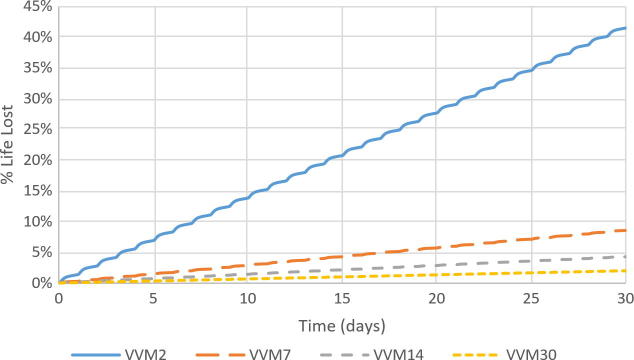
Loss of VVM life over 30 days using maximum observed vial warming (Setup 8).

These results demonstrate that while vials in all test setups were exposed to temperatures above 5 °C, 30 days of cyclic warming would not inevitably cause early conversion to endpoint for any category of VVM. The life lost during a month for all four categories of VVM using the worst-case vial temperature curve, from setup 8, is shown in Fig. 3, where the importance of vaccine heat stability by proxy of VVM is evident. The VVM7 curve in Fig. 3 shows a degradation of 8.56% over a month. In order for a VVM7 vial in the same location to reach endpoint during that month, it would have to be at least 91.44% decayed at the start of the month, a time period equivalent to 29.3 months at 5 °C. VVM14 and VVM30 categories were affected even less. A VVM2 vial in the same location might have reached endpoint early if it had been kept at a constant 5 °C for 3.5 months prior to the start of temperature cycling. The lifetime of a VVM2 at a constant 5 °C is only 5.9 months (using the middle of the VVM endpoint tolerance range, VVM2 life at 5 °C is 177 days, not 225 days), so its lifetime would be shortened by 2.4 months due to cooling water packs in the same refrigerator. This is well within the shelf life of prequalified vaccines, so extra caution would be necessary when evaluating the usefulness of this practice in a refrigerator containing low-stability vaccines (VVM2).

We investigated several factors that could influence the level of warming a vial would be exposed to, including the number of water packs cooling at a time, the arrangement and spacing of a fixed number of vials in the refrigerator, and the total number of vaccine vials in the refrigerator. From setup 1 to setup 2, the water pack load was increased from four to eight water packs, enough to fill one or two vaccine carriers. The average percentage life lost for the most sensitive category—VVM2—over a month increased 2.92%—from 19.98% to 22.90%—due to the increase in water pack load, and the maximum for any measured vial increased 7.33%. To investigate the impact of vial arrangement, ten vials were tested in identical arrangements on the floor and on a shelf in the refrigerator in setup 3 and setup 4. The average life lost increased 10.59% and the maximum increased 9.66% due to moving the vials from the floor to the shelf.

**Fig. 4:**
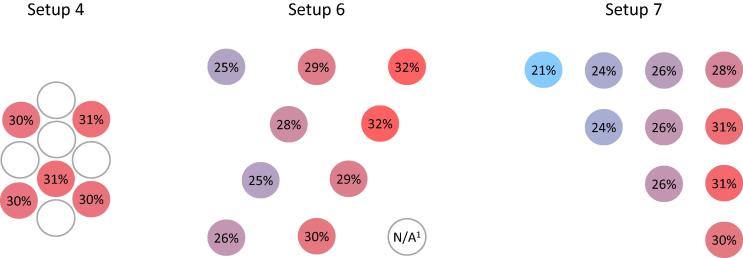
Heat map of vaccine vial monitor category 2 (VVM2) life lost in percentage over 30 days for three different test setups. Each setup contained 10 vials on a shelf and 8 water packs. Increasing the space between vials increased the range of VVM life lost. Vials closer to the water packs and farther from the walls lost more life. ^1^ The thermocouple in this location was discovered not submerged in water inside the vial; therefore, these data have been omitted.

The spacing of vials was tested by arranging ten vials close together in setup 4, then distributing them more loosely in setups 6 and 7. Results for the VVM2 category were similar for these tests, the largest difference among the three being a decrease of 3.62% in the average life lost from setup 4 to setup 7. However, there was an increase of 0.61% in the maximum life lost between the same tests despite the decrease in the average life lost, indicating that a larger spatial distribution leads to a wider range of life lost, due to varying proximity to the water packs (Fig. 4). In setup 7, the average life lost for the four vials closest to the water packs was 9.32% higher than the vial furthest.

To investigate the impact of vial load on vial warming, tests were run with 1 or 10 vials on a shelf in setups 5 and 4, respectively, and with 20 and 168 vials on the refrigerator floor in setups 2 and 8, respectively. The average calculated life lost across 10 vials for VVM2 in setup 4 was 4.16% lower than that for the single vial in setup 5, and the maximum single vial life lost in setup 4 was 3.81% lower than that for the single vial in setup 5 (Fig. 1).

Comparing 20 vials in setup 2 and 168 vials in setup 8, the average VVM2 percentage life lost across 30 days increased 3.15% from setup 2 to setup 8, but the maximum increased 9.72%, indicating that there was again higher variability in the larger vial load. This is evident when comparing the vial arrangements layer to layer: the average VVM2 percentage life lost decreased 1.51% from setup 2 to setup 8 when comparing only the bottom layer of vials on the refrigerator floor. The percentage life lost for the second layer decreased 0.25%. Only the top two layers of the setup 8 vial arrangement contributed to the overall increase in life lost from setup 2 to setup 8. This stratification across layers, as well as the temperature gradient due to proximity to the water packs seen in setup 7, demonstrates that location in the refrigerator has a higher impact on VVM life lost than the number of vials, though the number and location of vials in the refrigerator are dependent. However, as it might be expected, VVM category was shown to have a greater effect on VVM life than any of the other factors investigated here.

Some limitations of this study were that testing was only carried out in one refrigerator and that many tests were carried out with lower vial loads than would likely be found in a vaccine refrigerator in a low-resource setting. Other factors that would be valuable to study include the volume of vaccine in the vials, the impact of refrigerator air flows, and the impact of warming in different types of refrigerators. In addition, there is a lack of data on the impact of thermal cycling on the relationship between vaccine potency and VVM behavior within this temperature range. Another caution is that if the vaccines had not returned to equilibrium before cooled water packs were exchanged for additional warm ones, the thermal impact could intensify and it might take fewer cycles before the VVMs on the vaccines reached endpoint. One further concern regarding the use of VVMs as a proxy for vaccine life is the correlation between VVM decay and vaccine decay. VVMs do not provide direct information on stability [12] or potency [15] of a vaccine; however, their stated purpose is to indicate when the cumulative heat exposure has reached a preset limit, and to that end, they have been well validated at temperatures lower than 37 °C [16]. One study noted a lack of correlation between loss of vaccine potency and VVM performance at higher temperatures [17], but the warmest vial throughout our testing was well within the validated range.

## 4 Conclusions

Our study suggests that serious damage to vaccines cannot be assumed from a single excursion event or from repeated exposure to warm water packs in the same refrigerator over the course of a month. Several factors result in loss of VVM life due to warming, but the inherent heat stability represented by the different categories of VVMs was found to be most important; i.e., vaccines in vials with a VVM2 are most likely to be damaged from cyclic warming. The other important factor was location in the refrigerator, with the phase-change material-lined floor and walls of the testing refrigerator better at resisting warming of vials. In many situations, the practice of placing warm water packs in a vaccine refrigerator would not cause any serious loss of vaccines beyond that occurring at cold chain temperatures over time. Further research is necessary to define safe ratios of and distances between vaccine vials and warm water packs in varying refrigerators. If the decrease in cool life due to using cool water-packs is acceptable, and if there is extra space available in vaccine refrigerators, cooling water packs in a refrigerator with vaccines may be a beneficial practice in cold chain management. By using space in already installed and available equipment, this practice could reduce loss of vaccines due to freezing without additional cost. The data presented here and the process outlined for evaluating the effects of the practice are useful tools to support cold chain management and regulatory entities in making these decisions.
