# Supplementary material for: The effect of cool water pack preparation on vaccine vial temperatures in refrigerators
Source: Vaccine. 2018 Jan 2;36(1):128–33. doi: 10.1016/j.vaccine.2017.11.024 (PMC5736983; doi:10.1016/j.vaccine.2017.11.024)
Supplement: Supplementary data 1 [file mmc1.docx]

Appendix 1: Instrumented vial locations

| **Setup 1**  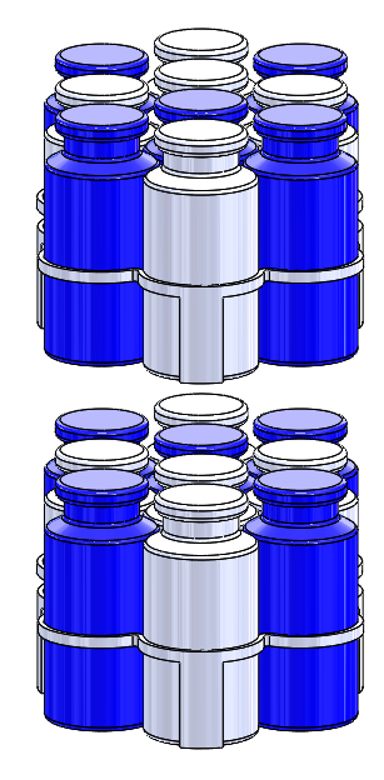 | **Setup 5**  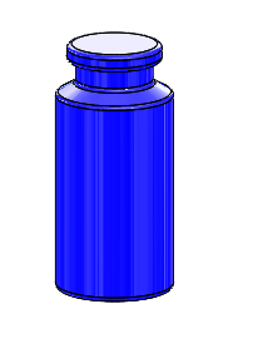 |
| --- | --- |
|  | **Setup 6**  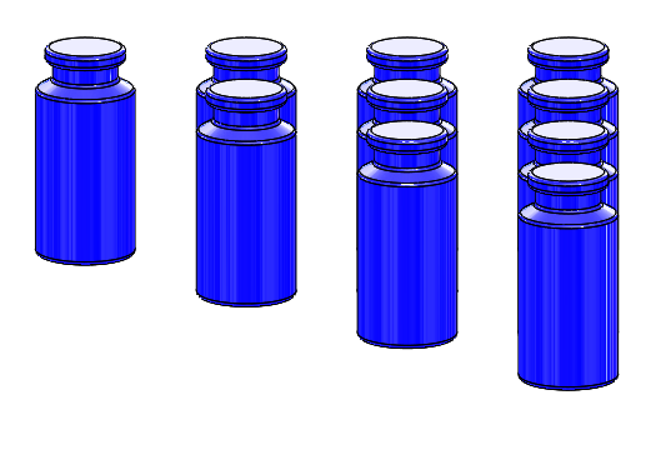 |
| **Setup 2**  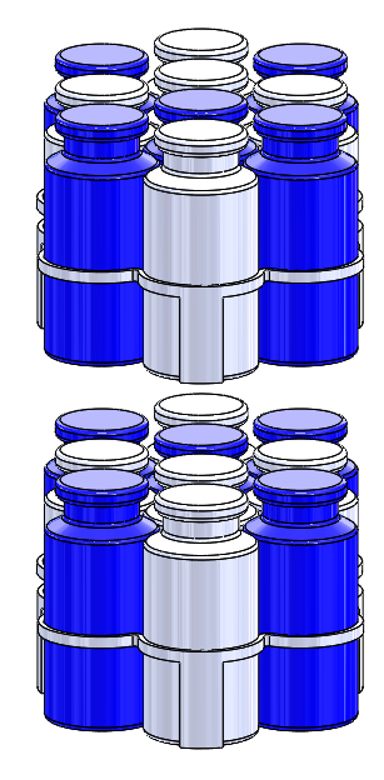 | **Setup 7**  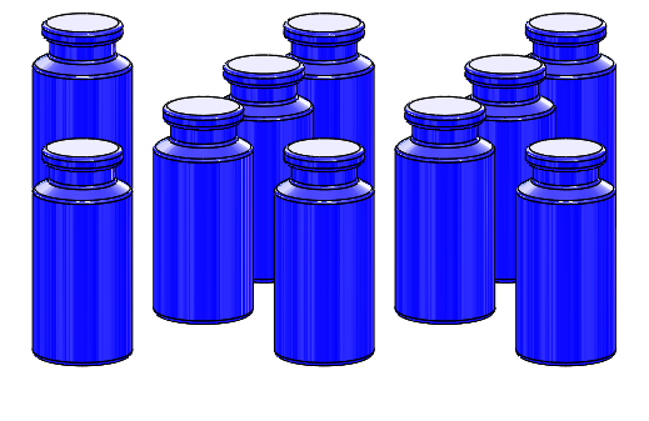 |
|  | **Setup 8**  **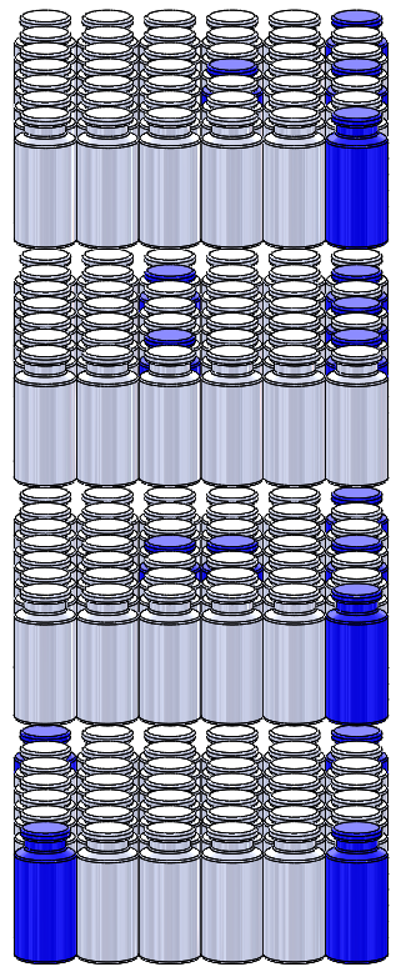** |
| **Setup 3**  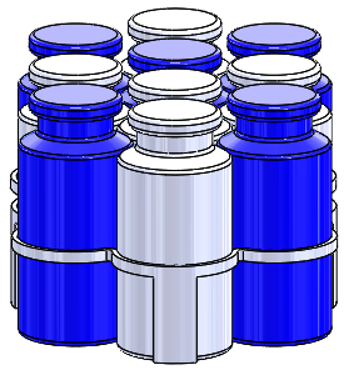 |  |
| **Setup 4**  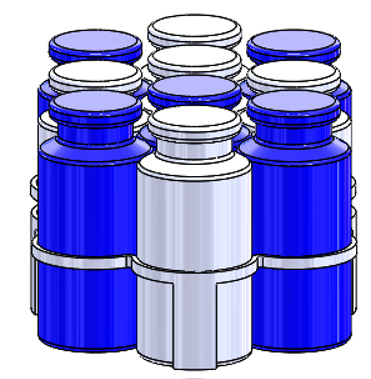 |  |

Dark blue indicates instrumented vials.
